# Efficacy evaluation of endolift laser for treatment of nasolabial folds and marionette lines

**DOI:** 10.1111/srt.13480

**Published:** 2023-09-27

**Authors:** Mohammad Ali Nilforoushzadeh, Maryam Heidari‐Kharaji, Tannaz Fakhim, Seyedeh Tina Hosseini, Shohreh Rafiee, Mohammadhasan Shahverdi, Niloufar Najar Nobari

**Affiliations:** ^1^ Skin and Stem Cell Research Center Tehran University of Medical Sciences Tehran Iran; ^2^ Skin Repair Research Center Jordan Dermatology and Hair Transplantation Center Tehran Iran; ^3^ Institut National de la Recherche Scientifique (INRS)‐Centre Armand‐Frappier Santé Biotechnologie (CAFSB) Laval, Québec Canada; ^4^ Department of Cellular and Molecular Biology Faculty of Advanced Science and Technology Tehran Medical Sciences Islamic Azad University Tehran Iran; ^5^ Department of Dermatology Rasool Akram Medical Complex Iran University of Medical Sciences Tehran Iran

**Keywords:** endolift laser, laser treatment, marionette lines, nasolabial folds, treatment

## Abstract

**Background:**

The nasolabial folds are the most marked sign of aging. Endolift laser was used for the treatment of nasolabial folds and marionette lines (one of the facial danger zones).

**Methods:**

Ten female subjects were engaged in this study. Patients underwent Endolift laser for nasolabial folds and marionette lines treatment. The efficacy of the Endolift technique on the nasolabial folds and marionette lines was evaluated by biometric assessment with Cutometer, Visioface, and the Skin Ultrasound Imaging system. Also, patient's satisfaction and blinded dermatologists’ assessment were assessed.

**Results:**

The Visoface results displayed that the Endolift laser treatment significantly declined the depth and area of the nasolabial wrinkles. The skin ultrasonography results reported that the epidermis and dermis density and thickness were significantly increased. Also, the cutometer outcomes showed that the Endolift laser treatment can increase skin elasticity. The results showed that a large number of patients were very satisfied with the technique.

**Conclusion:**

In conclusion, Endolift laser has an effective technique for decreasing the nasolabial folds, marionette lines, and improve the appearance of the face without any sever side effect. This technique does not need general anesthesia and recovery time.

## 1 INTRODUCTION

Aging leads to a decrease in a collagen production, increases the fat volume in the nasolabial folds lower portion, lose the nasolabial sulcus volume, and fold the upper cheek area,^1^ so protrude the nasolabial groove.^1^, ^2^ Also ageing lead to a reduction in skin thickness, that affects the biomechanical possessions of the skin.^3^ The aging is a natural process in the humans, but it can accelerate by several factors that cause advanced physiological and structural changes in the skin such as environmental pollution and sun exposure. These factors reduce elastic fibers and collagen and thus loss the skin elasticity.^4^, ^5^ Changes in the nasolabial folds are one of the primary features of face aging. These changes are due to morphologic changes of the muscle function and skin sagging.^6^ Nasolabial folds look like wrinkles, because both are shaped by folding of the dermis. Several aesthetic techniques are used for nasolabial rejuvenation like microneedle,^7^, ^8^ radiofrequency,^9^ and filler injection^10^, ^11^, ^12^, ^13^ but each method has its own risks. Ablative laser resurfacing by erbium: YAG (Er:YAG) laser and carbon dioxide laser is an effective method for rejuvenation of the skin but has probable side effects like, infection, prolonged erythema, and scarring.^14^ Sometimes, non‐ablative lasers for instance, neodymium: YAG (1320 nm) laser and fractional erbium glass (1550 nm) laser, are used together to reduce the ablative lasers side effects however they have less improvement and need more sessions for treatment.^15^, ^16^, ^17^, ^18^ Therefore, this study aimed to investigate the effect of Endolift laser therapy as a novel technique on nasolabial fold and marionette lines (one of the facial danger zones).

## 2 SUBJECTS AND METHODS

### 2.1 Subjects selection

In total, 10 healthy female and male patients suffering nasolabial fold and marionette lines were joined in this study. The age variety was from 25 to 58. Written informed consent forms were gained from all individuals for the investigations. Patients with coagulopathy, immunocompromised status, active infection, cancer, and autoimmune disease were excluded, pregnant patients were also excluded.

### 2.2 Study design and laser technique

**FIGURE 1:**
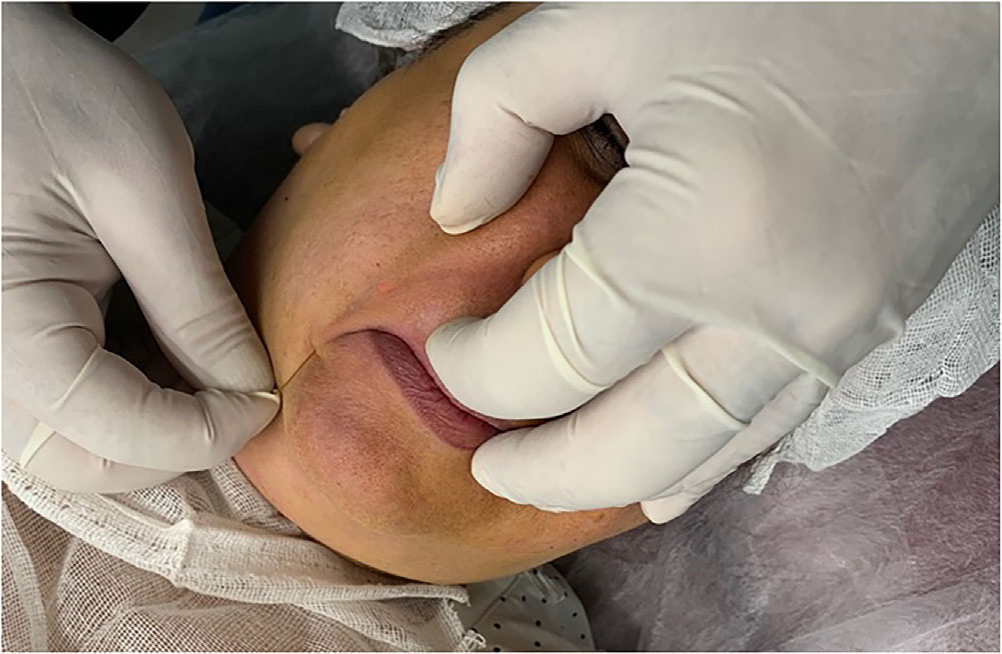
Endolift laser procedure.

Endolift™ (LASEMAR1500TM machine, Eufoton s.r.l.) was used in this study. A sterile set was prepared for each patient. A sterile set includes betadine galipot, injectable serum galipot, syringe containing injectable lidocaine for anesthesia, sterile gauze, sterile gloves, and sterile fibers for Endolift. The intervention area (perioral area) was cleaned using betadine and the target area was cleaned of betadine using injected serum. Injectable lidocaine (2% lidocaine) was used in the nasolabial area that was the site of Endolift fiber insertion in to the skin and the fiber movement area was numbed. After local anesthesia, the sterile fiber was inserted into the skin and the fiber was moved superficially in fanning motions to avoid temperature rise in the intervention area, the laser diode was induced at the same time (Figure 1). In this treatment method, the 300 μ fiber was used. To perform the procedure in this area, energy and time were reduced because this area is one of the facial danger zones. The intensity and the power of the device include; Pulse: 3, T on ‐T off: 25–75, shot number: 200–250 shots on each side of the face, total shots for the whole of the nasolabial area: 400–500. The intensity and power of the device were the same in all area of intervention and the patients received the treatment one time. The necessary care after treatment was explain to the patients. For the patients with the history of herpes, acyclovir tablets were prescribe and the antibiotic was prescribed to prevent possible infections after the procedure of 5 days. The possible side effects like infection, erythema, and swelling were noted and managed after treatment. This treatment method does not have recovery period or down time. The patients were followed for 3 months after the procedure and the they underwent biometry and skin analysis.

## 3 ASSESSMENT

### 3.1 Biometric evaluation

Before and after 3 months of the treatment, the patients biometric parameters were appraised by Cutometer, the Skin Ultrasound Imaging system (TPM, Germany; DUB Skin Scanner; 75 MHZ probe) and Visioface (Germany). The skin elasticity was measured by Cutometer with analyze of three factors R2, R5, and R7. The skin aging and elasticity were evaluated by R2 and R7 and it showed that they are the important parameters of Cutometer. R2 reported skin viscoelasticity, containing viscous deformation and R7 reported the biological elasticity, R5 reported net elasticity. The changes in the volume, depth, and area of the skin wrinkle were appraised by Visoface (multi‐probe adapter). The skin ultrasound imaging system is related to the dermis and epidermis diameter and density. The increase in the dermis density is associated with the increase in dermis collagen. The erythema was assessed by Mexameter MX 18 probe (data not shown).

### 3.2 Objective and subjective methods

Before and after 3 months, the treatment digital photographs were taken with identical camera (Nikon, 10.2 million pixels, Tokyo, Japan) settings. The clinical effects of the Endolift methods on the nasolabial fold and marionette lines were evaluated by objective and subjective methods. The objective method comprised three blinded dermatologist evaluations of photographs before and after laser treatment. The subjective method included patient's satisfaction. The outcomes were classified as follow: no satisfaction, slightly satisfied, moderately satisfied, and well satisfied.

### 3.3 Statistical analysis

Statistical analysis was performed by SPSS 15.0 statistical software (SPSS) in this study. A *P* value of less than 0.05 was noted statistically significant.

## 4 RESULTS

### 4.1 Biometric parameters changes results

**FIGURE 2:**
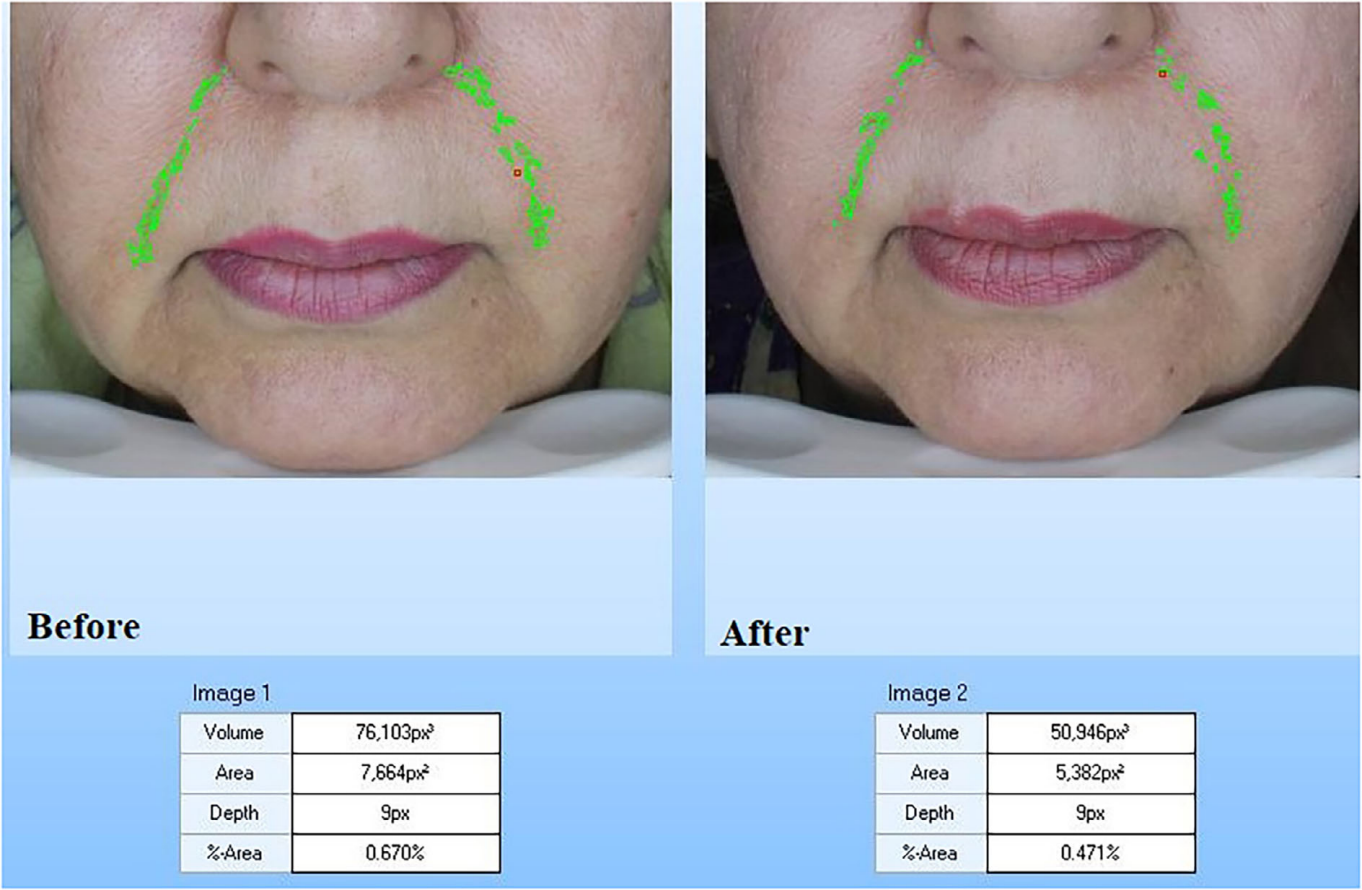
Visioface wrinkle analysis for case A: decrease in volume, area and depth of the nasolabial folds and marionette lines after treatment.

**FIGURE 3:**
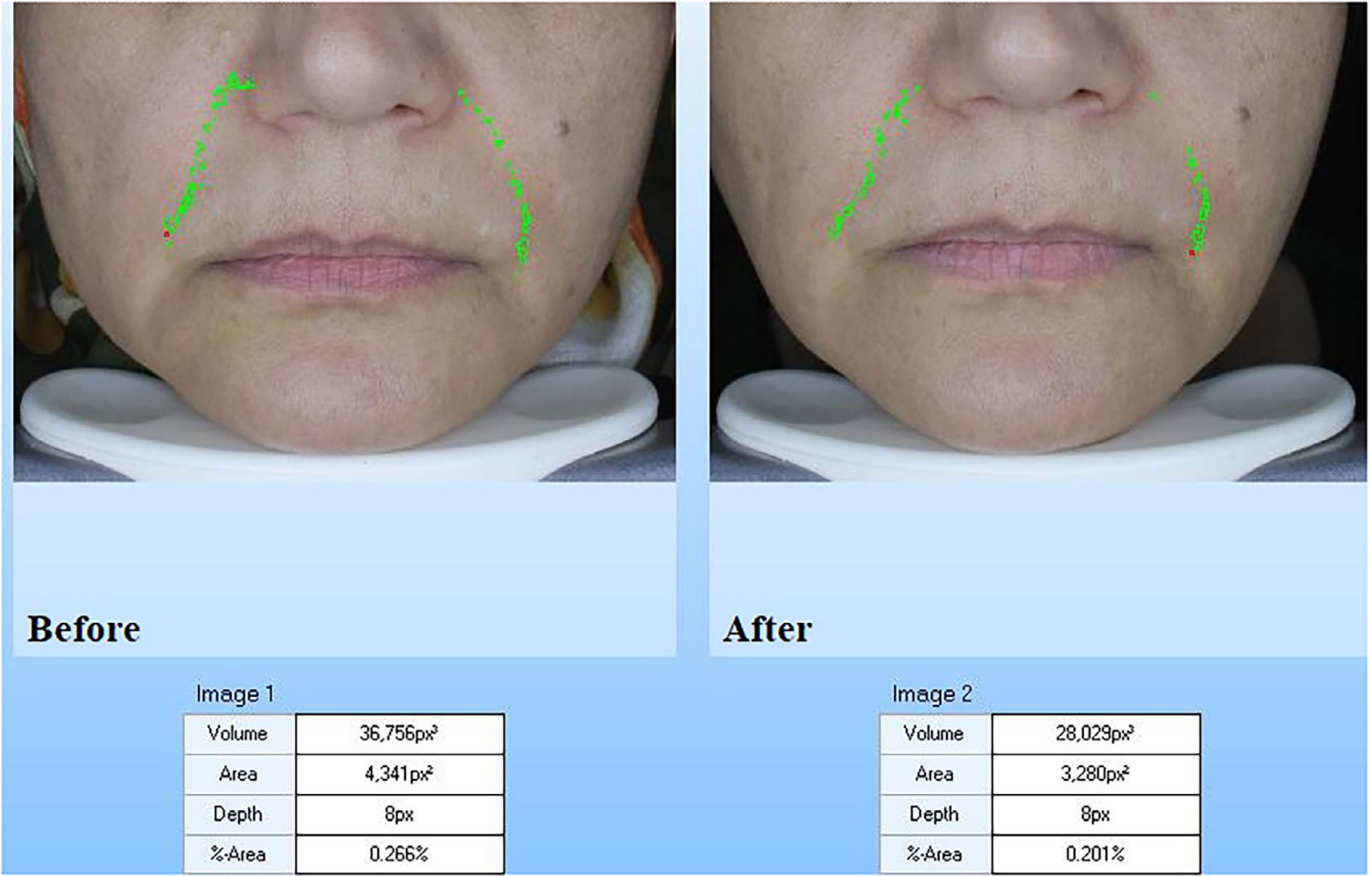
Visioface wrinkle analysis for case B: decrease in volume, area and depth of the nasolabial folds and marionette lines after treatment.

**FIGURE 4:**
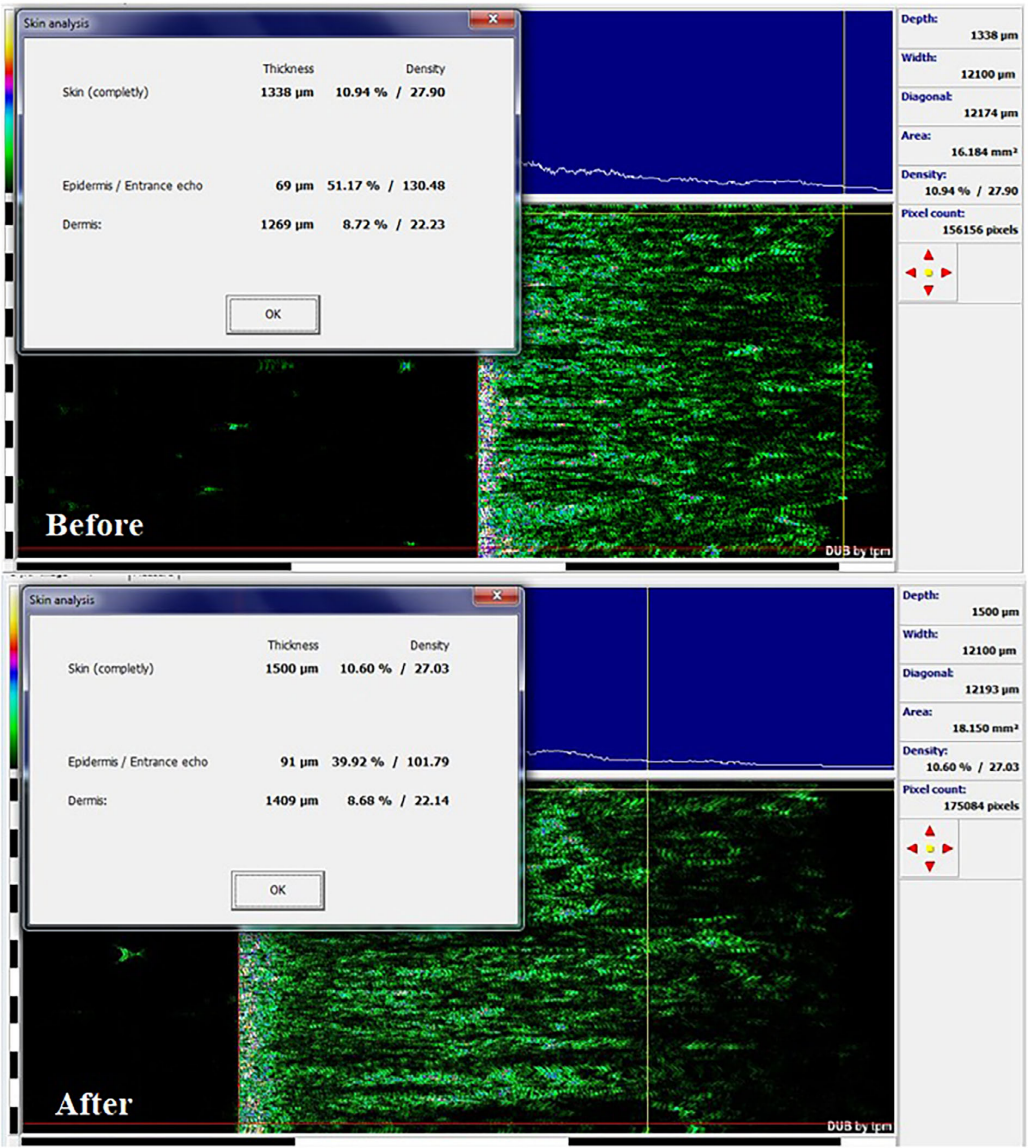
Skin analysis with ultrasonography for case A.

**FIGURE 5:**
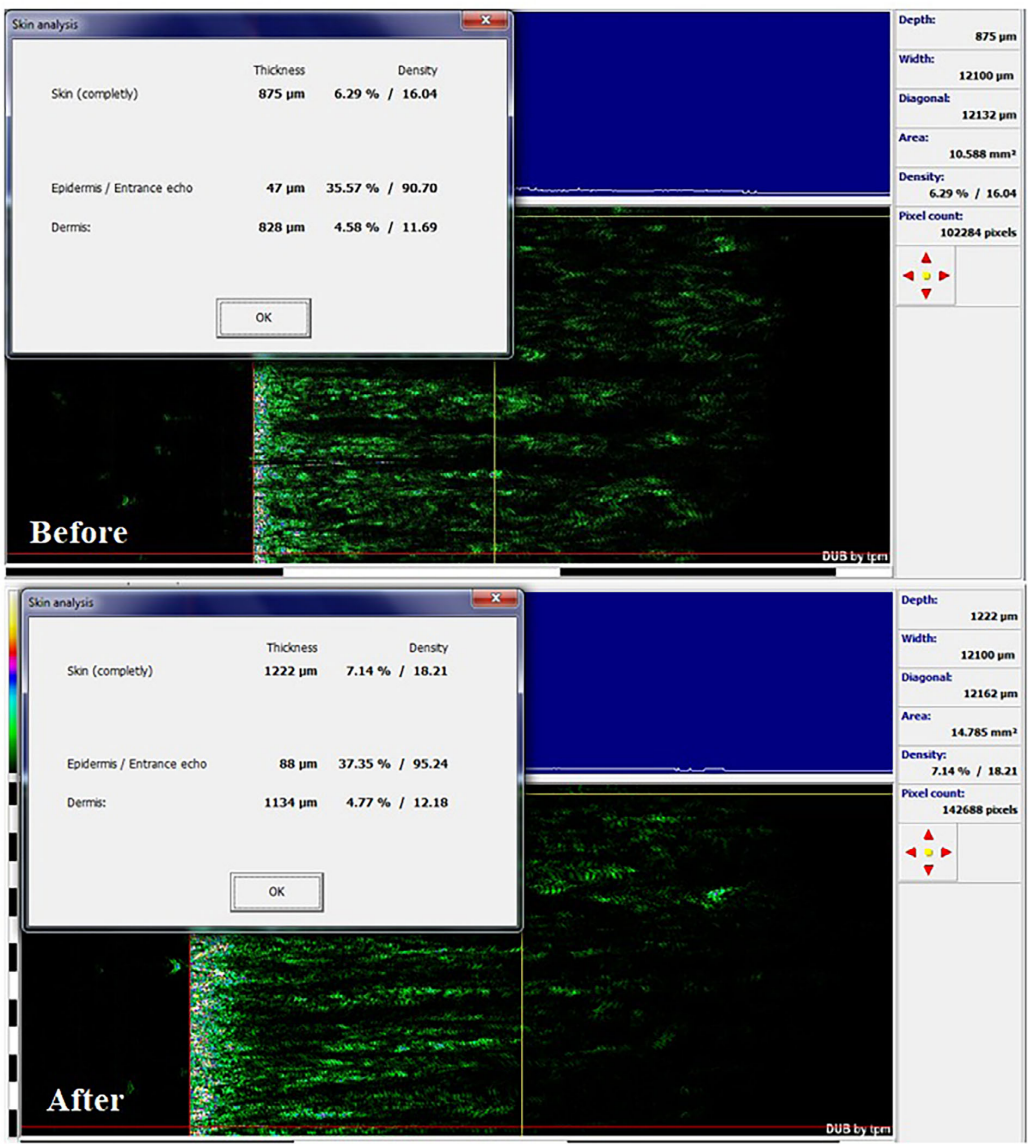
Skin analysis with ultrasonography for case B.

The biometric measurement results of all patients are shown in Table 1 as mean  ± SD. According to the Visioface results, the Endolift laser treatment significantly declined the depth and area of nasolabial wrinkle, and the percentages of change for volume and area were 26.78 ± 10.46 and 33.45 ± 9.73, respectively (*p* <0.05; Table 1). The results of Visioface for the cases A and B are shown in the Figures 2 and 3. Also, the skin ultrasonography results reported that the epidermis and dermis density and thickness are significantly increased (Table 1) and the percentages of change in epidermis thickness and density were 34.25 ± 11.02 and 28.19 ± 9.31 and for dermis thickness and density the percentages of change were 39.8 ± 11.12 and 43.75 ± 12.22 (*p* <0.05). The cases A and B skin ultrasound results are shown in Figures 4 and 5. The cutometer results showed similar results and the cutometer outcomes displayed that the Endolift laser treatment can increase skin elasticity (*p* <0.05) (Table 1). The percentages of change in R2, R5, and R7 were 25.32 ± 8.14, 26.13 ± 10.08, and 25.41 ± 6.26, respectively. The patients showed mild edema and erythema, after the treatment which was completely resolved after 1 or 2 days. The mexameter data showed no significant difference between the erythema before and after procedure. The patients were monitored for 3 months and no side effects were informed during the study.

**TABLE 1:** Comparing biometric characteristics of the skin before and 3 months after treatment.

	Measured values			
	Before	After	Percent change	*P* value
**Visioface**				
Wrinkle				
Volume (px^3^)	63.55 ± 20.1	46.36 ± 19.27	26.78 ± 10.46	<0.05
Area (%)	0.60 ± 0.17	0.4.33 ± 0.19	33.45 ± 9.73	<0.05
**Skin ultrasonography**				
Skin density (μm)	6.30 ± 2.12	8.71 ± 4.16	38.11 ± 9.21	<0.05
Skin thickness (μm)	875.12 ± 180.62	1138.25 ± 206.01	30.02 ± 10.13	<0.05
Epidermis density	35.13 ± 11.16	45.15 ± 12.12	28.19 ± 9.31	<0.05
Epidermis thickness	47.44 ± 15.11	63.11 ± 17.15	34.25 ± 11.02	<0.05
Dermis density	4.29 ± 4.29	6.14 ± 6.71	43.75 ± 12.22	<0.05
Dermis thickness	876.71 ± 160.07	1219.14 ± 166.13	39.8 ± 11.12	<0.05
**Density** ^a^				
R2	0.70 ± 0.12	0.88 ± 0.12	25.32 ± 8.14	<0.05
R5	0.52 ± 0.04	0.66 ± 0.16	26.13 ± 10.08	<0.05
R7	0.36 ± 0.02	0.45 ± 0.04	25.41 ± 6.26	<0.05

^a^ Density of the skin measured by cutometer.

### 4.2 Patients and physicians’ assessment results

Regarding patients’ satisfaction, a significant number of patients were well satisfied (*N* = 6) after treatment (Table 2) (*p* <0.05). The physician reported a significant satisfaction after Endolift laser treatment. The results are shown in Table 3 (*p* < 0.05).

**TABLE 2:** Patient satisfaction for nasolabial folds and marionette lines treatment 3 months after Endolift laser treatment. The *P* <0.05 was considered as statistically significant.

	Valid	Frequency	Percent	*P* value
After 3 months	No satisfaction	0	0	≤0.05
	Moderately satisfied	4	40	
	Well satisfied	6	60	
	Total	10	100	

**TABLE 3:** Physician assessment after treatment. The data was shown as mean ± SD.

Dermatologists	Physician satisfaction (%) (mean ± SD)	*P* value
Physician 1	80 ± 2.2	*P* < 0.05
Physician 2	78 ± 3.3	
Physician 3	75 ± 3.1	

## 5 DISCUSSION

In the past decades, there have been many requests for skin rejuvenation to achieve youthful looking skin. Nasolabial folds are one of the signs of aging that depends on several factors such as smoking, aging, and sun exposure. Nasolabial folds look like wrinkles, because both are shaped by folding of the dermis. Nasolabial folds and marionette lines are the most visible signs of aging in lower face likewise, they are very common at the age of under 30.^19^ Most of the patients would like nasolabial fold and marionette lines to be less visible. In this study, Endolift laser technique was performed for the nasolabial fold and marionette lines treatment. In this research study, the results were evaluated by biometric changed and patients satisfaction. Also, three blind dermatologist assessed the results. Our Visioface results displayed that the Endolift laser significantly decrease the area and depth of nasolabial fold and marionette lines. The skin ultrasonography results reported that the epidermis and dermis density and thickness were significantly increased. Also the cutometer outcomes displayed that the Endolift laser treatment can increase skin elasticity. In some previous published study, the effect of Endolift laser for treatment of forehead wrinkles, acne scars, upper eyelid, and eyebrow ptosis, arm and under abdomen fat, lower eyebag, skin laxity, jowl fat, were evaluated.^20^, ^21^, ^22^, ^23^, ^24^, ^25^, ^26^, ^27^, ^28^ Our results were similar to these studies results. According to these previous studies, Endolift laser can decrease the skin laxity and can tight the skin and also can decrease the facial wrinkles. To our knowledge, this is the first report of Endolift laser that show it is a safe and effective method to decrease the nasolabial fold and marionette lines. and improve the appearance of the face. This procedure does not require general anesthesia and recovery time. Various treatment methods have been used for nasolabial folds’ improvement comprising, botulinum toxin injection, dermal fillers, high intensity focal ultrasound (HIFU), fractional CO2 laser, and thread lifting.^29^ Sometimes, to gain a better cosmetic results, a combination of some treatment modalities is used. Laser skin resurfacing has still been considered the gold standard for skin rejuvenation. Howyda et al. reported that intraoral Er:YAG laser is effective treatment for nasolabial folds wrinkle.^30^ There are some studies studying the efficacy of intraoral fractional Er: YAG laser in improvement of nasolabial folds.^31^, ^32^ In this study we used Endolift laser for nasolabial fold treatment. The Endolift laser technique is non‐invasive method with don't need anesthesia, recovery time, and have no adverse effects, also it will tolerate with the patients. Endolift laser is leading to shrinkage and contraction of tissue which resulting in new collagen formation and collagen remodeling. The collagen is responsible for improvement of skin thickness, elasticity, and fill in wrinkles^33^, ^34^, ^35^, ^36^. So this technique can increase tissue tightness and improvement of nasolabial fold wrinkles. Endolift laser method gives a significant improvement in the patients, particularly those who did not prefer filler or surgery.

## 6 CONCLUSION

Endolift laser is an effective technique for decrease of the nasolabial folds and marionette lines (one of the facial danger zones) and improvement of the appearance of the face without any sever side effect. This technique does not need general anesthesia and recovery time.

### 6.1 Study limitation

Further studies are recommended to assess more numbers of patients and longer follow‐up periods.

## CONFLICT OF INTEREST STATEMENT

The authors declare that they have no conflict of interest.

## ETHICS STATEMENT

Informed consent was obtained from all the patients. All patients were provided with a complete description of the study design, purpose, and probable outcomes. All the patients were checked before and 6 months after the last session of treatment.

## Data Availability

The data that support the findings of this study are available from the corresponding author upon reasonable request.
